# Supplementary material for: Loneliness and cognitive decline among U.S. adults: A stratified analysis of the BRFSS
Source: PLoS One. 2026 May 8;21(5):e0339554. doi: 10.1371/journal.pone.0339554 (PMC13155599; doi:10.1371/journal.pone.0339554)
Supplement: Attachment — Submitted filename: Review.docx [file pone.0339554.s001.docx]

**Introduction**

The Introduction would benefit from revisions to improve clarity, logical flow, and conceptual grounding across paragraphs. Here are some minor and major points:

1. Sentence structure and flow (Lines 57–58):

The sentence “Defined as a distressing subjective state arising when perceived social connections are inadequate” is a fragment and should be rewritten as a complete sentence or integrated with the preceding sentence.

2. Conceptual clarity between loneliness and social isolation (Lines 59–62):

The Introduction moves abruptly from prevalence estimates of social isolation (e.g., “nearly one quarter of those aged 65 and older are socially isolated”) to stating that loneliness and social isolation are distinct, followed immediately by “the present study focuses on loneliness.” This transition should be smoother.

3. Psychosocial vs. physiological framing (Lines 63–65):

The phrase “Beyond its psychosocial toll” implies that psychosocial consequences of loneliness have already been discussed, but they are not described earlier in the paragraph. This contrast should be clarified or rephrased to maintain logical continuity.

4. Insufficient contextualization of subjective cognitive decline (Lines 69–70):

Subjective cognitive decline is introduced briefly via prevalence estimates but is not conceptually developed. Given that cognitive decline, dementia, and SCD are related but distinct constructs, the authors should provide additional background on what SCD indicates, what it predicts, and why it is important to study SCD separately—particularly in relation to loneliness.

5. Blurring of background and methods (Lines 85–87):

The description of the BRFSS SCD item reads as methodological detail and may be more appropriate for the Methods section, or should be reframed as background information motivating the study.

6. Limited justification for analytic “contributions” (Lines 88–94):

The stated contributions would benefit from clearer theoretical or empirical justification, including: why a dose–response relationship between loneliness and SCD is hypothesized, how causal language is justified given the cross-sectional design, and why associations are expected to differ by sex, age, and race/ethnicity.

7. Need for more specific hypotheses (Lines 96–98):

The hypotheses are broad and largely restate the analytic plan. More specific, theory-driven hypotheses would strengthen the Introduction and better align with the proposed contributions.

**Methods**

1. Clearer justification for the inclusion of several covariates:

In particular, variables such as health insurance type, metropolitan status, urbanicity, and language appear to be included without explanation beyond their availability in the dataset. The authors should clarify the conceptual or empirical rationale for adjusting for these factors (e.g., whether they are considered confounders, proxies for access to care or socioeconomic context, or related to reporting of loneliness or subjective cognitive decline). Providing this justification would help readers assess the appropriateness of the adjustment set and improve interpretability of the findings.

Also, depressive symptoms/depression is a well-known factor of loneliness and cognition. Please include it as covariate.

2. Age categorization (18–64 vs. ≥65) should be justified, as this choice may obscure heterogeneity within older age groups.

**Results/Discussion**

1. Use of “chronic loneliness” or “persistent loneliness” with cross-sectional measurement:

The Results section refers to “chronic loneliness or persistent loneliness,” but loneliness appears to be measured at a single time point without information on duration or persistence. Without longitudinal or retrospective data on how long loneliness has been experienced, it is unclear how chronicity is being operationalized. The authors should clarify what they mean by “chronic” in this context or revise the terminology to avoid implying temporal persistence that is not measured.

2. Ambiguous terminology (e.g., “frequently married”):

The phrase *“frequently married”* is unclear and potentially misleading.

3. Overinterpretation of baseline group differences in a very large sample:

The statement that baseline characteristics *“highlight pronounced socioeconomic, psychosocial, and demographic differences”* may overstate the substantive meaning of these differences. Given the very large sample size, statistically significant differences are expected even when effect sizes are small. The authors should be cautious in interpreting these differences and consider reporting or referencing effect sizes, or rephrasing to avoid implying meaningful group separation based solely on statistical significance.

4. Dose-Response Interpretation:

While the graded pattern across loneliness categories is visually compelling, the interpretation of a strong “dose–response relationship” warrants additional verification. Given the very large sample size, statistically significant differences across all categories may reflect high power rather than substantively meaningful contrasts. I encourage the authors to supplement pairwise comparisons with additional robustness checks, such as reporting effect sizes (e.g., absolute risk differences), conducting a formal test for trend, or evaluating potential non-linear or threshold effects (e.g., contrasting high-frequency loneliness vs. lower-frequency groups). This would strengthen confidence that the observed pattern reflects a meaningful dose–response relationship rather than precision alone.

Also, the dose–response framing implicitly assumes a monotonic relationship in which increasing frequency of loneliness is uniformly more harmful. However, prior loneliness research suggests that occasional or moderate loneliness may be normative and not necessarily detrimental, whereas adverse effects may emerge only beyond certain thresholds of frequency or severity. The authors may wish to consider alternative characterizations of the pattern (e.g., threshold or non-linear effects) or to discuss why a linear or monotonic interpretation is theoretically justified in this context.

Clarifying these issues would strengthen the interpretation of the findings and avoid overstatement of dose–response effects based solely on categorical contrasts in a cross-sectional, high-powered dataset.

5. Causal and temporal language not supported by the data:

Phrases such as “detrimental impact,” “consequences,” and “heightened vulnerability” imply a causal or longitudinal interpretation. Given the cross-sectional design and single-time measurement of loneliness and subjective cognitive decline, the authors should avoid language suggesting effects or consequences and instead describe these findings as differences in associations or predicted probabilities.

6. Outcome Interpretation and Terminology:

The results and discussion section incorrectly refers to the outcome as “predicted cognitive function”. However, the outcome is subjective cognitive decline, operationalized as a binary self-report of worsening confusion or memory over the past 12 months. This measure does not capture cognitive function or performance per se, but rather perceived cognitive change. Even though SCD has been associated with objective cognitive impairment and dementia risk in prior studies, describing results as differences in “cognitive function” overstates what is measured. The authors should revise the language throughout to refer to predicted probability of subjective cognitive decline or self-reported cognitive difficulties, rather than cognitive function.

7. Interpretation of Sex Differences in High Loneliness:

The explanation offered for the observed sex difference among individuals reporting persistent loneliness is not well aligned with the results or the constructs measured in this study. While the authors report that women who are *always lonely* have higher predicted probabilities of subjective cognitive decline than men, the proposed explanation—that women’s family and social networks have weakened—invokes objective social network characteristics that were neither measured nor analyzed. This is particularly problematic given the authors’ earlier emphasis that loneliness and social isolation are related but distinct constructs.

As currently written, this explanation appears speculative and not directly supported by the data. The authors should either provide empirical evidence linking sex differences in loneliness-related cognitive complaints to network disruption, or reframe this interpretation more cautiously (e.g., in terms of differential psychological, emotional, or perceptual responses to loneliness among women). Clarifying this distinction would improve conceptual consistency and strengthen the discussion.

8. Racial/Ethnic Differences and Cultural Interpretation:

The discussion of racial/ethnic differences raises an important point about potential cultural variation in how loneliness relates to cognition, but this section would benefit from further development. Statements referring to “stronger family and community networks,” “culturally distinct interpretations of loneliness,” and “differences in reporting SCD” are plausible but remain speculative and are not sufficiently elaborated.

Given that the authors invoke “complex cultural differences” to interpret these findings, it would strengthen the discussion to expand on what these differences may entail, drawing more explicitly on prior literature (e.g., cultural norms around emotional expression, familism, stigma, or differential meaning of loneliness and cognitive complaints across racial/ethnic groups).
